# Mechanisms and Regulation of Nonsense-Mediated mRNA Decay and Nonsense-Associated Altered Splicing in Lymphocytes

**DOI:** 10.3390/ijms21041335

**Published:** 2020-02-17

**Authors:** Jean-Marie Lambert, Mohamad Omar Ashi, Nivine Srour, Laurent Delpy, Jérôme Saulière

**Affiliations:** Unit CNRS 7276 - INSERM U1262—Limoges University, 2 rue du Docteur Marcland, 87025 Limoges, France; jean-marie.lambert@unilim.fr (J.-M.L.); mohamad-omar.ashi@unilim.fr (M.O.A.); nivine.srour@mail.mcgill.ca (N.S.); jerome.sauliere@unilim.fr (J.S.)

**Keywords:** Immunoglobulin (Ig), nonsense-mediated mRNA decay (NMD), nonsense-associated altered splicing (NAS), B lymphocytes, plasma cells

## Abstract

The presence of premature termination codons (PTCs) in transcripts is dangerous for the cell as they encode potentially deleterious truncated proteins that can act with dominant-negative or gain-of-function effects. To avoid the synthesis of these shortened polypeptides, several RNA surveillance systems can be activated to decrease the level of PTC-containing mRNAs. Nonsense-mediated mRNA decay (NMD) ensures an accelerated degradation of mRNAs harboring PTCs by using several key NMD factors such as up-frameshift (UPF) proteins. Another pathway called nonsense-associated altered splicing (NAS) upregulates transcripts that have skipped disturbing PTCs by alternative splicing. Thus, these RNA quality control processes eliminate abnormal PTC-containing mRNAs from the cells by using positive and negative responses. In this review, we describe the general mechanisms of NMD and NAS and their respective involvement in the decay of aberrant immunoglobulin and TCR transcripts in lymphocytes.

## 1. Mechanisms of Nonsense-Mediated mRNA Decay (NMD)

**Figure 1:**
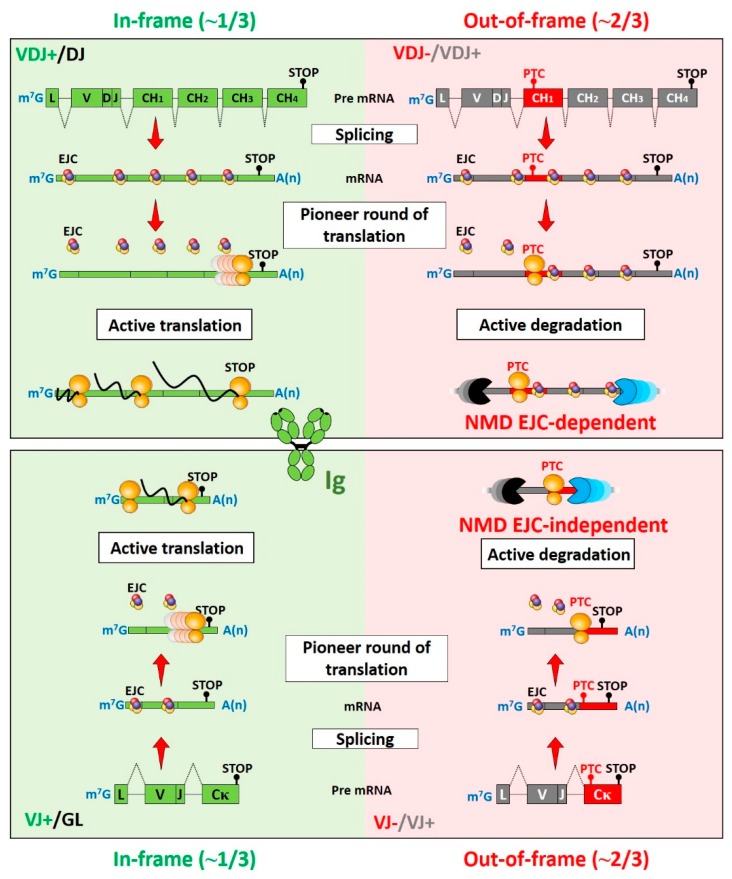
NMD pathways involved in the degradation of nonproductive Ig mRNAs. A multi-protein exon junction complex (EJC) is deposited 20–24 nucleotides upstream of each exon–exon junction during splicing of most transcripts. These EJCs remain associated with mRNAs until the first round of translation. Complete EJC removal after ribosomal reading serves as a licensing step for active translation. In contrast, the presence of premature termination codons (PTCs) >50 nt upstream of the last exon–exon junction precludes the removal of downstream EJCs and triggers accelerated degradation of PTC-containing mRNAs. Imprecise V(D)J junctions can generate around 1/3 of productive (P) and 2/3 of nonproductive (NP) V(D)J-rearranged immunoglobulin (Ig) alleles [82,83]. NP Ig heavy chain (IgH) mRNAs represent good EJC-dependent NMD substrates because PTCs are located within the VDJ exon or in the first constant exon (CH1). A PTC position within the CH1 exon (red) is depicted for NP IgH transcripts (upper right). By contrast, the appearance of PTCs on NP IgL mRNAs does not respect the canonical position rule for EJC-dependent NMD. Indeed, PTCs that appear after an out-of-frame VJ junction are located either in the last constant exon (lower right), or near the last exon–exon junction (not depicted). The degradation of NP IgL mRNAs involves an EJC-independent NMD pathway that senses abnormally long distances between the PTC and the polyA tail. GL: germline; V(D)J+: productive V(D)J rearrangement; V(D)J−: nonproductive V(D)J rearrangement.

The NMD pathway ensures accelerated degradation of premature termination codon (PTC)-containing mRNAs to avoid the synthesis of truncated proteins with potentially deleterious effects for cells [1,2,3]. PTCs can arise in a variety of ways. For example, the most obvious source is random nonsense and frameshift mutations introduced by errors during DNA replication, RNA transcription, or splicing [4]. Another source of PTCs is programmed DNA rearrangements that occur during lymphocyte development to generate the antigen receptor repertoire. In two thirds of cases, these rearrangements lead to the generation of frameshift mutations and consequent downstream PTCs, which in turn activate NMD [5] (Figure 1). NMD also regulates 5% to 15% of physiological mRNAs that harbor upstream open reading frames (uORF), introns downstream of normal translation termination codons, or mRNAs with selenocysteine codons [4,6,7,8,9,10,11]. It has been estimated that about 30% of inherited human diseases are due to the presence of PTCs or frameshifts that induce nonsense codons in mRNAs [12,13], and NMD is also involved in human cancers [14,15].

The central NMD factor in human cells is the up-frameshift protein 1 (UPF1), which is an RNA-dependent ATPase and ATP-dependent RNA helicase [16,17,18,19]. This protein unwinds RNA in the 5′ to 3′ direction, probably to eliminate ribonucleoproteins (RNPs) from the targeted mRNAs and thereby favor their degradation by other NMD factors [20,21]. When a ribosome stalls on a PTC, UPF1 associates with the eukaryotic release factors 1 and 3 (eRF1 and eRF3) and with the suppressor with morphogenic effect on genitalia 1 (SMG1) kinase (a protein kinase related to phosphatidylinositol 3-kinases). This forms the SMG1–UPF1–eRF1–eRF3 (SURF) complex with SMG8 and SMG9, which inhibits the kinase activity of SMG1 [22,23,24]. UPF1 is activated by its SMG1-dependent phosphorylation upon the dissociation of SMG8 and SMG9 [25]. Recently, it was shown that DExH-box helicase 34 (DHX34) acts as a scaffold for SMG1 and UPF1, facilitating its phosphorylation during NMD and thus allowing the conversion of SURF to the decay-inducing complex (DECID) [26,27]. The exon junction complex (EJC) is a multiprotein complex composed of a core comprising the eukaryotic translation initiation factor 4A3 (eIF4A3), cancer susceptibility candidate 3 (CASC3 or MLN51 standing for metastatic lymph node 51), and the heterodimer composed of RNA-binding motif protein 8A (RBM8A or Y14) and mago nashi homolog (MAGOH or Barentsz in Drosophila) [28]. The EJC is loaded 20–24 nucleotides (nt) upstream of roughly 80% of exon–exon junctions [29,30,31,32]. During the pioneer (or first) round of translation, all EJCs are displaced from the CBC (cap-binding complex)-bound mRNAs by the ribosomes in absence of PTCs [33,34,35,36]. If a ribosome stalls on a PTC >50 nt upstream of the last exonic junction marked by an EJC, NMD is activated (mechanisms detailed below). Despite this pioneer round model, it has also been shown that eIF4E (eukaryotic initiation factor 4E)-associated mRNAs (i.e., during active translation) can be subjected to NMD as efficiently as those associated with CBC [33,36]. 

The EJC core is a scaffold platform that loads additional NMD factors including UPF3 in the nucleus and UPF2 in the cytoplasm [37,38]. Two paralogs of UPF3 exist: UPF3A and UPF3B (also known as UPF3X). Recently, it was demonstrated that UPF3A and UPF3B control NMD by acting in an antagonistic manner [39]. Indeed, UPF3B is important for NMD, in contrast to UPF3A which acts as a NMD inhibitor by sequestering UPF2 [39]. UPF3B binding to the EJC results in a direct interaction between UPF2 and UPF1 that in turn activates its helicase activity to allow mRNA unwinding and protein remodeling [16]. It was previously shown in an in vitro translation termination system that free UPF3B is also capable of interacting with release factors to delay translation termination at the PTC by sterically blocking stop codon recognition [40,41]. UPF1 phosphorylation leads to the recruitment of three additional NMD factors, SMG5, SMG6, and SMG7 [42,43,44,45]. SMG6 protein, which contains an active PIN (PilT N-terminal) domain, is recruited to the NMD complex, leading to the endonucleolytic cleavage of targeted mRNAs in vicinity of the PTC [46,47,48]. In contrast, SMG5 and SMG7, which do not possess active PIN domains [47], form a heterodimer on the NMD target that respectively recruits decapping enzymes (DCP2 and DCP1A) and deadenylases (CCR4-NOT) [49,50,51]. PNRC2 (proline-rich nuclear receptor coactivator 2) can also bind SMG5, the phosphorylated form of UPF1 and DCP1A [49,50,52,53]. This finally leads to removal of the 5′ cap and 3′ polyA tail to degrade NMD targets by exonucleases in a 5′ to 3′ direction by XRN1 and in a 3′ to 5′ direction by the exosome [54,55]. NMD is therefore a very important pathway for the regulation of mRNA levels in cells, but its molecular mechanisms and the components of the NMD machinery are yet not fully characterized. For example, five additional NMD factors (ngp-1, npp-20, aex-6, pbs-2, and noah-2) were recently identified by genome-wide RNAi screening in nematodes and their molecular functions in this mRNA surveillance process are under investigation [56]. Moreover, NMD can also act as an antiviral process by impeding viral infections and viruses can inflect NMD [57,58,59,60,61,62,63,64]. Indeed, it has been shown that UPF1, SMG5, and SMG7 (all these NMD factors are described in more detail below) restrict SFV (Semlicki Forest Virus) replication in human cells [57]. Moreover, Tax protein from T-lymphotrophic virus type 1 inhibits NMD by interacting with UPF1 and INT6 (which is required for efficient NMD) [65,66].

The model described above is a general overview of the NMD pathway in mammalian cells, but several studies performed in yeast and invertebrates have revealed alternative NMD pathways. For example, in the budding yeast *Saccharomyces cerevisiae*, only the UPF factors and a DEAD-box helicase (called Fal1p), highly homologous to eIF4AIII, are present [67]. The prevailing model of NMD in yeast is called the “faux 3′UTR”, where ribosomes stalled on the PTC fail to interact with the appropriate 3′UTR-bound proteins [68,69]. Indeed, PABPC1 (PolyA Binding Protein C1), a natural 3′UTR RNA-binding protein, can interact with eukaryotic release factor 3 (eRF3) to ensure efficient translation termination [22,23,68,70,71]. If this interaction is not possible (for example in the case of a long 3′UTR generated by the presence of a PTC), the surveillance complex is assembled leading to mRNA decay [68,72]. Moreover, in mammals, some examples of EJC-independent NMD have been reported for immunoglobulin (Ig) transcripts which acquire PTCs at high frequencies during B lymphocyte development [73,74] (Figure 1). In addition, NMD is mainly EJC-independent in invertebrates such as *Drosophila melanogaster* and the *Caenorhabditis elegans* nematode [75,76]. In the fission yeast *Schizosaccarhomyces pombe*, NMD is splicing-dependent but does not require the EJC [77]. Recently, it was also demonstrated that NMD is EJC-independent in the early branching eukaryote protozoan *Tetrahymena thermophila* ciliate [78]. Alternative NMD routes have also been described that can be driven by the EJC with differential EJC co-factor requirements. For example, reduced abundance of the EJC co-factor RNPS1 is correlated with low NMD efficiency [79,80]. Moreover, NMD is inhibited when a PTC is closed to the translation initiation AUG codon in the case of short ORFs (open reading frames) [81]. In conclusion, NMD is a complex cellular process involving different pathways to ensure the efficient degradation of mRNAs harboring PTCs and to regulate the levels of physiological transcripts essential for cellular homeostasis.

## 2. Fluctuations of NMD Efficiency during B-Cell Development

The error-prone V(D)J recombination process frequently generates PTCs in lymphocytes [5,84]. NMD has been extensively studied in T cells, in which very efficient degradation of PTC-containing TCR-β mRNAs has been documented [5,85,86,87,88,89]. Accordingly, perturbation of T-cell development has been observed in NMD-deficient mice [90,91].

In recent decades, several laboratories, including ours, have contributed to the understanding of how PTC-containing Ig mRNAs are degraded by NMD [5,73,92,93,94,95,96,97]. As exemplified for Ig heavy (IgH) and light (IgL) chain genes (Figure 1), the imprecise nature of V(D)J recombination generates ~1/3 of in-frame and ~2/3 of out-of-frame V(D)J junctions. Nonproductive V(D)J junctions can lead to the appearance of PTCs at the end of the variable (V) exon or in the downstream adjacent constant exon. For IgH mRNAs that contain several constant exons, the presence of PTC in the V or CH1 exon elicits EJC-dependent NMD. By contrast, PTC-containing IgL mRNAs do not conform to the −50 nt boundary rule and harbor PTCs close to or within the last constant exon. Therefore, many B-lineage cells express PTC-containing Ig mRNAs that can activate both EJC-dependent and -independent NMD modes [82].

**Figure 2:**
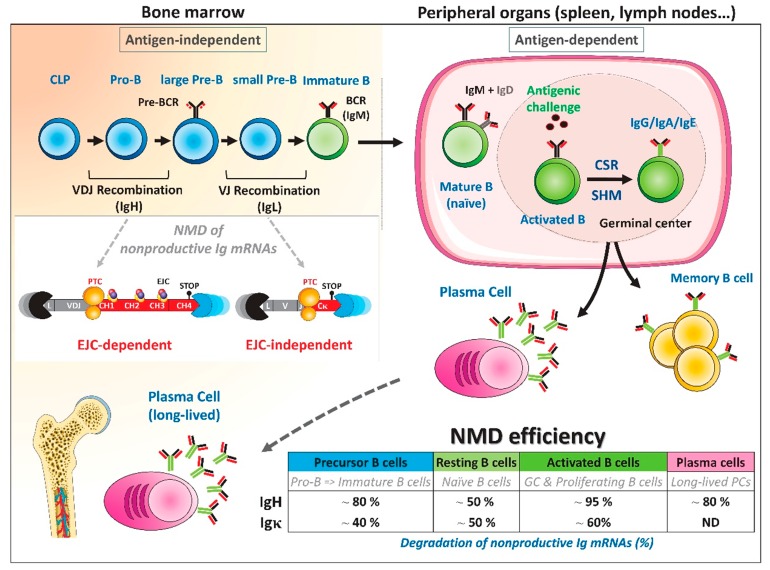
NMD assessment during B-cell development. Early B-cell development takes place in bone marrow through antigen-independent stages. B cell precursors undergo a first round of DNA rearrangements, between VH, DH, and JH segments located in the 5′ region of the Ig heavy (IgH) chain locus. V(D)J recombination is initiated by DH to JH rearrangements at the pro-B stage and followed by VH to DJH recombination. At the large pre-B stage, a productive (P) VDJ rearrangement encodes membrane Igµ chains that can associate with surrogate light chains to form the pre-B cell receptor (pre-BCR). Provided appropriate pre-BCR signaling, V to J rearrangements will be initiated at Ig light chain loci in small pre-B cells, leading to the expression of a functional BCR at the immature B cell stage. Once positively selected, mature B cells migrate to the periphery. Upon antigen stimulation, B cells proliferate in germinal centers (GCs) and further diversify their Ig repertoire through class switch recombination (CSR) and somatic hypermutations (SHM). Activated B cells can then differentiate into memory B cells or plasma cells (PCs). PCs will return to bone marrow niches in which they can survive for several years. Mouse models have been used to quantify the extent of degradation of nonproductive (NP) Ig mRNAs during B-cell development [94,97]. Fluctuations of NMD efficiency are depicted in the lower right table. A very active EJC-dependent NMD is elicited for NP IgH mRNAs, with almost complete disappearance of PTC-containing IgH mRNAs in activated B cells [97]. In contrast, the magnitude of EJC-independent NMD is far lower for NP IgL mRNAs, with only 60% of degradation in activated B cells [94]. ND: not determined.

To evaluate the downregulation of PTC-containing IgH mRNAs during B-cell development, we developed a mouse strain in which one IgH allele was rendered nonfunctional by inserting a “frameshift-inducing V exon” (frV) between JH and Cµ [97]. After VDJ recombination, the inactivating extra-V exon is spliced between the VDJ and CH1 exons and induces frameshifts at both acceptor and donor splice sites. According to the number of nts inserted at the VDJ junction, PTCs appear either in the VDJ, the frV, or in the constant CH1 exon. Hence, the position of PTCs on “frV knock-in” IgH mRNAs elicits EJC-dependent NMD regardless of the nature of the VDJ junction. In heterozygous IgH^frV/+^ animals, the expression of productive VDJ-rearranged wild-type (wt) IgH alleles drives normal B-cell maturation, while NMD efficiency can be easily assessed by quantifying the amount of PTC-containing “frV knock-in” IgH mRNAs. After treatment with drugs classically used to inhibit NMD, such as cycloheximide (CHX) or Wortmannin (wort), we observed that the NMD efficiency fluctuated during B-cell development (Figure 2). In bone marrow B-lineage cells, including precursors and plasma cells, treatment with NMD inhibitors raised the level of PTC-containing IgH mRNAs ~5-fold, indicating that approximately 80% of nonproductive IgH transcripts were degraded by NMD. By contrast, the extent of downregulation dropped to ~50% in naïve mature B cells. Interestingly, NMD efficiency was greatly increased after B-cell activation, with almost complete NMD degradation (~95%) of PTC-containing IgH mRNAs. Moreover, a positive correlation between RNA splicing and NMD degradation of PTC+ IgH transcripts was observed [97]. This was in agreement with previous findings by Gudikote and colleagues indicating that the strength of splice sites on PTC-containing TCR-β transcripts determines the extent of NMD. Indeed, these authors showed that TCR-β transcripts have strong splice sites and are rich in exonic splicing enhancer (ESE) sequences, which recruits splicing-enhancing factors such as serine/arginine-rich (SR) proteins. These motifs allow strong PTC-mediated downregulation, probably by EJC deposition modulation [88].

IgL transcripts are good models to study the magnitude of EJC-independent NMD because nonproductive VJ junctions cause the appearance of PTCs that do not fulfill the −50 nt boundary rule [82,95]. The downregulation of PTC-containing IgL mRNAs has been assessed during B-cell development using a mouse model that freely accumulates random IgL rearrangements in B cells without any selection for a functional BCR [94]. The “LMP2A” strain has been described previously and harbors the replacement of JH segments by the Epstein–Barr virus *LMP2A* gene [98]. The signaling cascade induced by LMP2A protein mimics the B-cell receptor (BCR) tonic signal and induces B lymphocytes to differentiate in the absence of a normal BCR. Using this model, we found that approximatively 40% to 60% of PTC-containing Igκ mRNAs were downregulated by a CHX sensitive NMD mode (Figure 2). Consistent with the degradation of PTC-containing IgH mRNAs, we also observed that B-cell activation promoted NMD of nonproductive Igκ mRNAs, compared to resting B cells (Figure 2). It was noted that downregulation of nonproductive Igκ mRNAs was lower than that observed for PTC-containing IgH mRNAs [94,97]. These physiological observations were in agreement with previous data from Mühlemann’s laboratory, obtained using minigene constructs [73,93]. Indeed, Bühler and colleagues found that Ig mRNAs harboring a PTC downstream of the −50 nt boundary rule were less efficiently degraded than those with an upstream PTC [93]. Therefore, the presence of EJC can enhance NMD efficacy. These authors also demonstrated that EJC-independent NMD relied more on the 3′UTR length [73]. Accordingly, knock-down of the EJC core protein eIF4AIII did not affect NMD efficiency, but the distance between the termination codon (TC) and the polyA tail was an important feature for EJC-independent NMD [73]. Long 3′UTRs could impair the local interaction between ribosomes stalled to TC and polyA binding protein (PABP) within the mRNP.

In conclusion, B-cell activation is accompanied by strong degradation of nonproductively rearranged Ig mRNAs involving both EJC-dependent and -independent modes of NMD. The reinforcement of NMD controls the amount of truncated Ig, which could impede the efficacy of immune responses.

## 3. Nonsense-Associated Altered Splicing (NAS)

As another cellular RNA surveillance pathway limiting the amount of PTC-containing mRNAs, NAS can be activated in response to nonsense mutations [99,100,101]. This mechanism increases the level of alternatively spliced RNA isoforms that have skipped offending PTCs. Thus, NAS is considered a positive post-transcriptional way to eliminate the PTC-containing exons from a transcript. The molecular mechanisms leading to NAS activation are still poorly understood, even if several studies have attempted to provide insights into this process [102,103,104]. Knockdowns of several key NMD factors including UPF1, UPF2, UPF3a, UPF3b, and SMG1 showed that only UPF1 is necessary for the alternative splicing and decay of PTC-containing mRNAs [100,103,105]. This is the only common feature with NMD where UPF1 is the central effector of this pathway. The RNA helicase eIF4AIII, one the core proteins of the EJC, is not involved in NAS, suggesting that the EJC deposited on exon–exon junctions as a splicing “mark” is not necessary for NAS [103]. Altogether, these findings suggest that NAS and NMD might be mechanistically different, sharing only the key NMD factor UPF1. Because the translation of PTC-free alternatively spliced mRNAs can generate shortened polypeptides with potentially deleterious functions [106], NMD and NAS sometimes display opposite functions with regard to the production of truncated proteins.

Two classes of NAS have been characterized. First, class-I NAS depends on the disruption of *cis*-splicing elements like ESE by all types of nonsense, silent, or missense mutations [103,107,108]. ESE stimulates splicing by acting as a binding site for splicing factors like some SR proteins [109,110]. Consequently, mutations in ESE, as is the case with a PTC, can favor exon skipping of this PTC-containing exon. Second, class-II NAS is triggered by disruption of the reading frame in the transcript. NAS of T-cell receptor β (TCR-β) transcripts is a well-characterized example of such class-II NAS [103]. Indeed, a frame-dependent NAS has been reported by Wilkinson’s laboratory in studies of mouse TCR-β minigenes in human cells [101,105]. However, studies done by Muhlemann’s laboratory did not reveal any correlation between synthesis of the alternatively spliced mRNA and truncation of the coding region [104]. These conflicting reports have rendered the effects of NAS on PTC-containing exons controversial.

**Figure 3:**
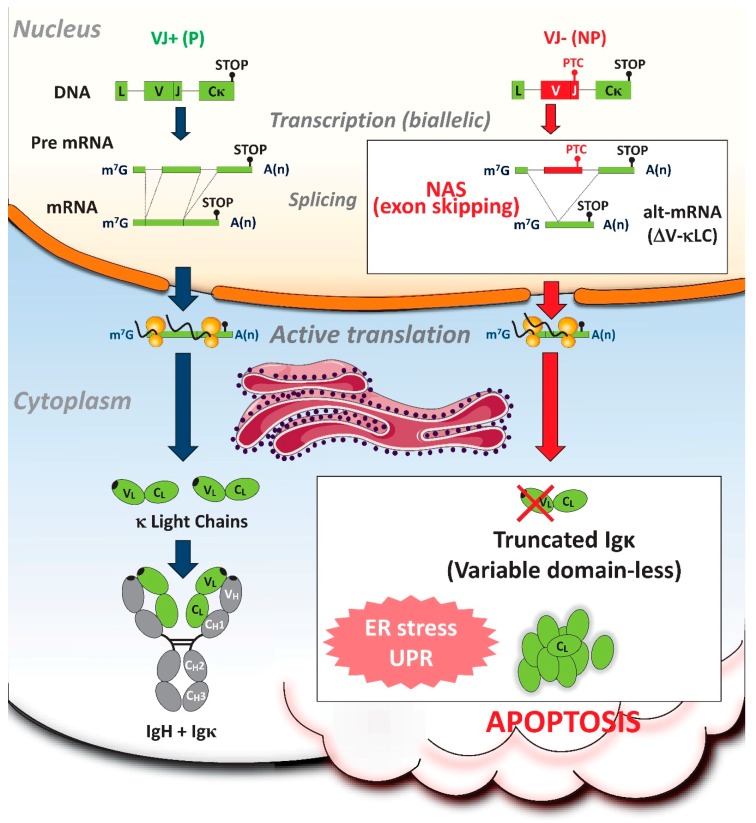
Consequences of NAS on Igκ transcripts in antibody-secreting plasma cells. Because most B-lineage cells harbor nonproductive (NP) Ig gene rearrangements [82,83], the boost of Ig gene transcription accompanying their plasma cell (PC) differentiation generates considerable amounts of nonsense Ig transcripts. In PCs with biallelic (VJ+/VJ−) Igκ rearrangements, the presence of premature termination codons (PTCs) activates NAS with skipping of the variable (V) exon. Remarkably, alternative V-less κ light chain (ΔV-κLC) mRNAs encode truncated Ig chains (lacking V-domain) which induce PC apoptosis through exacerbated ER stress and unfolded protein response (UPR) [106]. By eliminating numerous PC clones with biallelic Igκ rearrangements, in an antigen-independent mode, this novel truncated Ig exclusion (TIE) checkpoint reveals that NP Ig alleles can sometimes be drivers, rather than passengers. Altogether, the production of harmful truncated Ig provides evidence for deleterious NAS activation, confirming the assumption that this RNA surveillance process is highly risky and purposeless.

The NAS pathway is activated during splicing of nonproductively rearranged Igκ transcripts and provokes V exon skipping (Figure 3) [94,106]. Out-of-frame Vκ to Jκ rearrangements result in nonsense codons affecting either the 3′-end of the V exon (V^PTC^) or the last Cκ exon (C^PTC^) [95]. At the DNA level, V^PTC^ and C^PTC^ junctions are highly similar and often exhibit a single nt difference within the CDR3 sequences. To distinguish between class-I (ESE disruption) or class-II (disruption of reading frame) NAS for Igκ transcripts, the levels of alternatively spliced κ light chain mRNAs lacking V exon (ΔV-κLC) were measured in B-lymphoid cell lines transfected with minigene constructs mimicking nonproductive VκJκ5 junctions from both the V^PTC^ and the C^PTC^ class. This analysis revealed high levels of ΔV-κLC mRNAs in V^PTC^-expressing cells, whereas such transcripts were extremely rare in C^PTC^-expressing cells. These data strongly argue for a reading-frame-dependent class-II NAS that relies on PTC recognition within the skipped V exon, but not within the downstream Cκ exon. Accordingly, bioinformatic analyses performed using RESCUE-ESE Web Server did not reveal any differences in ESE sequence predictions [94]. Therefore, the NAS observed for Igκ transcripts most likely belongs to class-II NAS.

## 4. NAS of PTC-containing Ig RNAs during PC Differentiation

Our laboratory recently examined the magnitude of NAS during B-cell development and PC differentiation in vivo using the previously mentioned IgH^frVκ/+^ mice harboring an additional PTC-containing V exon on one IgH allele [111]. This model facilitates the quantification of NAS because Ig heavy chains can be produced after skipping of the PTC-containing extra-V exon. Interestingly, NAS of PTC-containing IgH RNAs was much more pronounced in PCs compared to B cells. The analysis of IgH transcription in different B and PC populations also revealed that the boost of Ig gene transcription accompanying PC differentiation correlated with high levels of NAS [111]. On one hand, alternative splicing is closely correlated to the rate of RNA polymerase II elongation and exon skipping is preferentially observed for highly transcribed genes [112,113]. On the other hand, Ig genes are localized in transcription factories that authorize cooperation with super-enhancers to increase Ig gene transcription in PCs [114]. Thus, the biallelic hyper-transcription of Ig genes in PCs strengthens NAS of nonproductively rearranged Ig RNAs [111].

While NMD ensures efficient degradation of nonproductive Ig mRNAs, activation of NAS can lead to the production of truncated Ig with V-domain deletions (Figure 3). A recent study by Srour and colleagues revealed the impact of truncated Ig chains produced after V exon skipping in PCs. Interestingly, the production of V-domain-less κ light chains induced endoplasmic reticulum (ER)-stress-associated apoptosis in antibody-secreting cells [106]. This novel “truncated-Ig exclusion” (TIE) checkpoint dampens PC differentiation by eliminating cells expressing nonproductive V^PTC^ Igκ alleles (Figure 3). Therefore, a risky NAS with skipping of PTC-containing V exons can eliminate PCs harboring biallelic Ig gene rearrangements. Remarkably, this TIE checkpoint reduces the magnitude of humoral responses independently of the classical constraints related to antigen specificity.

## 5. Concluding Remarks

Development of Ig repertoire diversity is achieved by DNA recombination between V, D, and J segments and imprecision at the VDJ junctions. A collateral effect of this random process is the generation of out-of-frame rearrangements associated with PTCs that could produce aberrant truncated Ig proteins with potential deleterious effects. Therefore, mRNA quality control mechanisms are very important processes that diminish the amount of PTC-containing Ig mRNAs. NMD recognizes these abnormal mRNAs as targets and degrades them by several complex mechanisms including EJC-dependent and independent pathways. EJC is the splicing mark deposited by the spliceosome on exon–exon junctions. If an EJC is located downstream of a PTC, NMD is activated by a complex array of interactions between the ribosome, NMD-associated factors, and EJC components. Alternatively, EJC-independent NMD can cause the degradation of aberrant Ig mRNAs by the absence of a physiological mRNP context at the 3′end of transcripts (i.e. long 3′UTRs). NAS is also involved in the targeting of mRNAs harboring PTCs by the exclusion of offending PTC-containing exons by alternative splicing. Even if to date some features have been characterized in the regulation of PTC-containing Ig mRNA levels, more experiments are still required to determine the molecular mechanisms responsible for NAS activation.

Exon skipping events eliminating the V exon can be induced by nonsense codons (NAS) or by using antisense oligonucleotides (ASO). Recent evidence has suggested that ASO-mediated Ig exon skipping can be easily achieved by targeting 5′ or 3′ splice sites on V exon pre-mRNAs [111]. Provided optimal drug delivery in bone marrow PC niches, the use of ASO to force the production of toxic truncated Ig chains should represent an attractive therapeutic approach. As a personalized strategy, ASO-mediated V exon skipping should provoke selective killing of PC clones in patients with monoclonal gammopathies (e.g., multiple myeloma, AL-amyloidosis, etc.).
